# Supplementary material for: Differential Dynamics and Roles of FKBP51 Isoforms and Their Implications for Targeted Therapies
Source: Int J Mol Sci. 2024 Nov 16;25(22):12318. doi: 10.3390/ijms252212318 (PMC11594789; doi:10.3390/ijms252212318)
Supplement: Supplementary file 1 [file ijms-25-12318-s001.zip › ijms-3199040-supplementary.pdf]

## Supplementary data

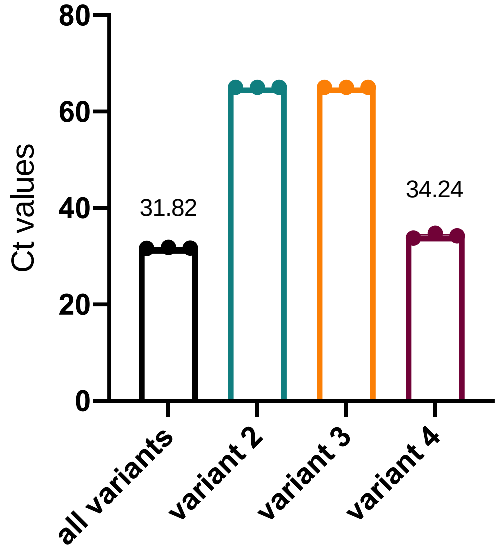


**Figure S1** – Plot of CT values of FKBP5 variants 1, 2, 3 and 4. Only variants 1 and 4 are detected in HeLa cells with CT means of 31.82 and 34.24 respectively). CTs of variants 2 and 3 are set by default at 65 as they are below the detection threshold.

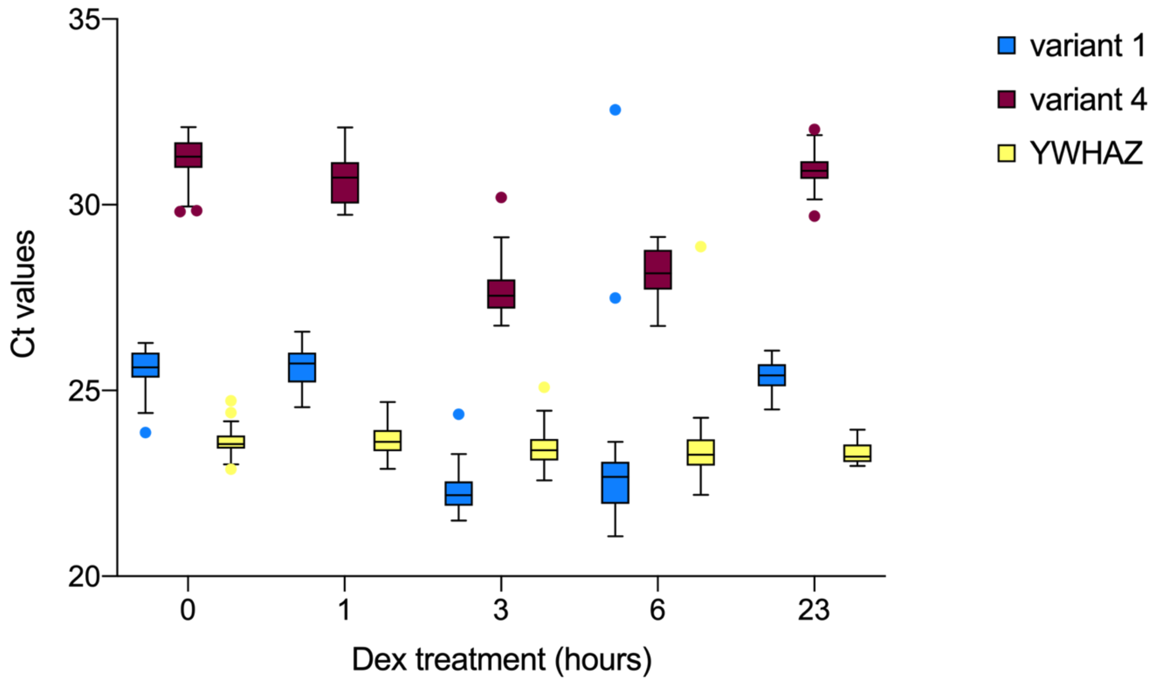


**Figure S2** – Plot of CT values of FKBP5 variant 1 and 4, and housekeeper gene YWHAZ. Variants 1 and 4 show a Dex-dependent expression, while YWHAZ is stable across the

time course. The plot highlights a notably higher expression of variant 1 compared to variant 4.

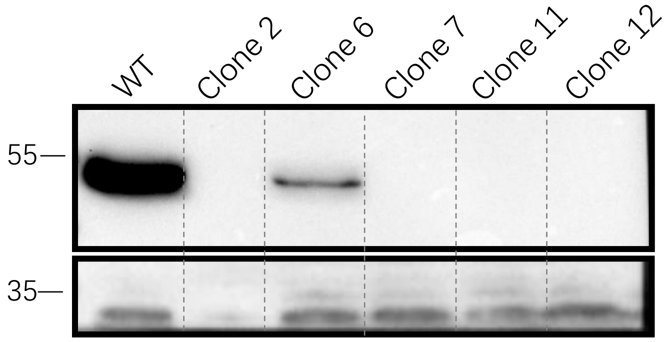


**Figure S3 –** HeLa KO clones selection: Western blot of selected clones generated via CRISPR-Cas as described in the method section. The upper part of the image shows FKBP51 isoform 1 detected by Abcam ab46002; the lower part shows FKBP51 isoform 2 detected by custom made antibody. Clones 2 and 12 were selected respectively as “full KO” and “isoform 1 KO”

**ruler:**

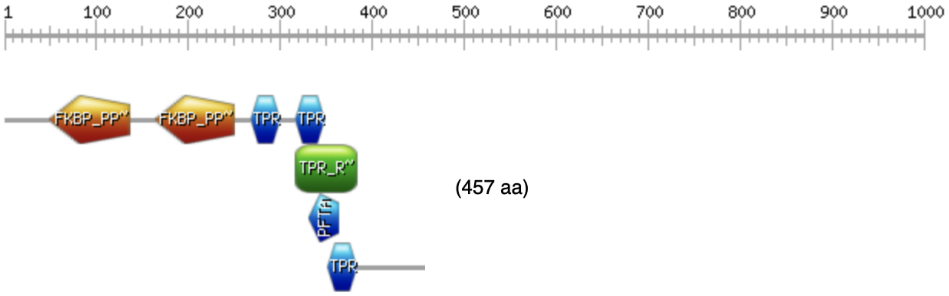


sp-Q13451-FKBP5_HUMAN (sp-Q13451-FKBP5_HUMAN )

(457 aa)

PS50059 **FKBP_PPIASE** *FKBP-type peptidyl-prolyl cis-trans isomerase domain profile* :

**50 - 138:** **score =** 30.133

GDKVYVHYKGKLSNGKKFDSSHDRNEPFVFSLGKGQVIKAWDIGVATMKKGEICHLLCKP
EYAYGSAGSLPKIPSNATLFFEIELLDFK

**Predicted feature:**

| | | | | |
|---|---|---|---|---|
| DOMAIN | 50 | 138 | PPIase FKBP-type | [condition: none] |

**165 - 251:** **score =** 19.620

GATVEIHLEGRCG-GRMFDCRD-----VAFTVGEGEdhdIPIGIDKALEKMQREEQCILY
LGPRYGFGEAGKPkFGIEPNAELIYEVTLKSFE

**Predicted feature:**

| | | | | |
|---|---|---|---|---|
| DOMAIN | 165 | 251 | PPIase FKBP-type | [condition: none] |

PS50005 **TPR** *TPR repeat profile* :

**268 - 301:** **score =** 5.723 [confidence level: R]

AAIVKEKGTVYFKGGKYMQAVIQYGKIVSWLEME

**317 - 350:** **score =** 9.529

LAAFLNLAMCYLKLREYTKAVECCDKALGLDSAN

**351 - 384:** **score =** 10.119

EKGLYRRGEAQLLMNEFESAKGDFEKVLEVNPQN

PS50293 **TPR_REGION** *TPR repeat region circular profile* :

**317 - 384:** **score =** 18.389

LAAFLNLAMCYLKLREYTKAVECCDKALGLDSANEKGLYRRGEAQLLMNEFESAKGDFEK
VLEVNPQN

PS51147 **PFTA** *Protein prenyltransferases alpha subunit repeat profile* :

**332 - 365:** **score =** 4.769 [warning: hit with a low confidence level (-1)]

EYTKAVECCDKALGLDSANEKG-LYRRGEAQLLMN

**ruler:**

sp-Q13451-2-FKBP5_HUMAN (sp-Q13451-2-F KBP5_HUMAN )

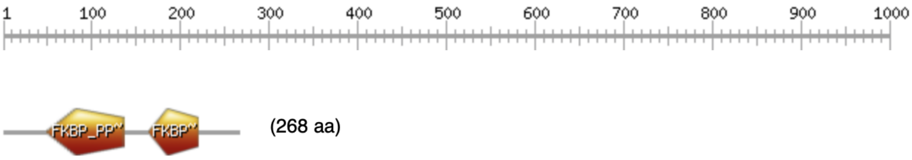


(268 aa)

PS50059 **FKBP_PPIASE** *FKBP-type peptidyl-prolyl cis-trans isomerase domain profile* :

**50 - 138:** **score =** 30.133

GDKVYVHYKGKLSNGKKFDSSHDRNEPFVFSLGKGQVIKAWDIGVATMKKGEICHLLCKP
EYAYGSAGSLPKIPSNATLFFEIELLDFK

**Predicted feature:**

DOMAIN 50 138 PPIase FKBP-type [condition: none]

**165 - 221:** **score =** 10.487

GATVEIHLEGRCG-GRMFDCRD-----VAFTVGEGEdhdIPIGIDKALEKMQREEQCILY
LGP-----------------------------

**Predicted feature:**

DOMAIN 165 221 PPIase FKBP-type [condition: none]

hits by profiles with a high probability of occurrence: [1 hit (by 1 profile) on 1 sequence]

Upper case represents match positions, lower case insert positions, and the '-' symbol represents deletions relative to the matching profile.

**ruler:**

sp-Q13451-2-FKBP5_HUMAN (sp-Q13451-2-F KBP5_HUMAN )

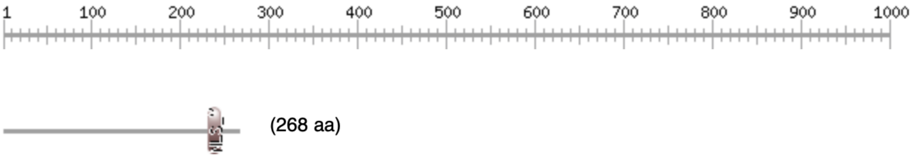


(268 aa)

PS50079 **NLS_BP** *Bipartite nuclear localization signal profile* :

**232 - 246:** **score =** 3.000 [warning: hit with a low confidence level (-1)]

KKNWSRLPLS--KRREP

**Figure S4** – Prosite results for FKBP51 isoforms 1 and 2
